# Healthcare professionals’ perspectives on the implementation and purposefulness of a new alcohol recovery Navigator role in the North East of England – preliminary qualitative findings

**DOI:** 10.1016/j.puhip.2025.100661

**Published:** 2025-09-30

**Authors:** Michael Cave, Ryan Swiers, Domna Salonen, Sarah Hulse, James Crosbie, Amy O'Donnell, Katherine Jackson, Emma-Joy Holland, Floor Christie-de Jong

**Affiliations:** a Newcastle City Council, Newcastle, United Kingdom; b Faculty of Health Sciences & Wellbeing, University of Sunderland, Sunderland, United Kingdom; c South Tyneside and Sunderland NHS Foundation Trust, United Kingdom; d Population Health Sciences Institute, Newcastle University, Newcastle, United Kingdom; e North East North Cumbria Integrated Care Board, United Kingdom; f School of Medicine, Faculty of Health Sciences, University of Sunderland, Sunderland, United Kingdom

**Keywords:** Alcohol recovery care, Patient Navigator, Qualitative, Staff perspectives, Normalisation process theory

## Abstract

**Objectives:**

This study aimed to explore clinicians’ perspectives in a single acute hospital regarding the introduction and perceived impact of an Alcohol Recovery Navigator role. The role was implemented within a hospital setting in North-East England to improve uptake of treatment in the community post-discharge and thereby help to reduce alcohol-related repeat admissions.

**Study design:**

A qualitative study was conducted.

**Methods:**

Semi-structured interviews were conducted with hospital clinicians (n = 8) recruited via purposive and snowball sampling. Interviews were transcribed verbatim, analysed using thematic analysis, with themes subsequently mapped onto Normalisation Process Theory constructs: coherence, cognitive participation, collective action, and reflexive monitoring.

**Results:**

Participants reported high levels of knowledge and understanding (coherence) of the Alcohol Recovery Navigator role and valued having this service as part of patients’ recovery pathway. Staff appeared committed to engaging with the role (cognitive participation), which was perceived to have aided implementation and embedding of the role into patient care pathways. Participants were able to make the role work (collective action) by building relationships with hospital staff and patients to improve continuity of care. Staff appraisal (reflexive monitoring) observed increased engagement from patients with Navigators and perceived that the role contributed to patients making changes towards better health.

**Conclusion:**

Participants’ perspectives support the continued provision of the Alcohol Recovery Navigator role. Implementation was viewed to have been successful, with Navigators imperative in bridging the gap between hospital and community care. Future research is required to assess the effectiveness of the wider programme.

## 1 Introduction

Reducing alcohol related harm is a global public health priority [1]. Patterns of high alcohol consumption is the third leading risk factor for premature death and disability worldwide and in 2019, 2.6 million deaths were attributable to alcohol consumption [1]. l In the UK, the number of alcohol-specific deaths has risen significantly since before the COVID19 pandemic. A record high of 10,473 alcohol-specific deaths were registered in 2023, 832 more than in 2021, a rise of 38.5 % in the space of four years [2]. During the same period, in England alone, 280,747 hospital admissions were recorded where the main reason was attributable to alcohol [3]. Alongside these acute health harms, alcohol use can contribute to a range of wider social and economic challenges that extend beyond individual harms. Alcohol is a risk factor that can adversely impact families and communities, including increased risk of domestic violence, significant emotional and psychological impact on children, and higher risk of child substance use [[4], [5], [6]]. The North-East of England has particularly high levels of alcohol consumption associated with harm and dependency, compared to other English regions [7], with 39 % of men and 18 % of women estimated to be consuming alcohol at high-risk levels [8]. From 2021 to 2022, the North-East had the highest rate of alcohol-related hospital admissions in England [9]. In the North-East, cost profiles across NHS healthcare, crime, the wider economy and social services amount to £1.49 billion overall, equivalent to £562 per head, compared with the national average of £485 [10]. As a region, the highest proportion of households experiencing socioeconomic disadvantage is the North-East and seven of the 12 North-East local authorities are ranked in the 30 % most deprived upper-tier authorities across England according to the Indices of Multiple Deprivation 2019 [11], indicating substantial health inequalities. Alcohol related harm is unequally and unfairly impacting those living in relative deprivation compared to economically advantaged individuals [12]. It is also important to consider how intersecting factors, such as gender, ethnicity, age, disability, and sexual orientation, may further shape these inequalities.

The NHS Long Term Plan (2019) set out to support hospitals with the highest rate of alcohol dependence-related admissions by establishing Alcohol Care Teams (ACTs) [13]. ACTs primarily provide specialist expertise and interventions for alcohol-dependent patients and those presenting with acute intoxication or other alcohol-related complications, attending emergency departments (ED), or being admitted as inpatients. ACTs decrease acute hospital admissions, readmissions, and mortality rates. They enhance the quality and efficiency of alcohol care, incorporating 11 key components that are evidence-based and cost-effective, one of the recommendations includes integrated care pathways [14]. However, focused improvement efforts are necessary for ACTs to achieve success and provide continuous person-centred care, as emphasised in the NHS long-term plan, thereby enhancing outcomes for this vulnerable group [15]. Recent findings highlight over 20 % of completed alcohol withdrawal admissions result in readmission within 30 days, strong predictors to readmission include unstable housing and high comorbidity pointing to the need for better co-ordination of community support and discharge planning [16]. In particular, there is a lack of uptake of aftercare treatment in the community from those who are repeatedly admitted to hospital.

The 2025 NHS updated plan continues to support alcohol related harm, highlighting the need to focus on prevention over treatment with emphasis on supporting community-based care and reducing health inequalities [14]. The navigator role supports the NHS goals to reduce re-admissions, improve continuity of care and tackling the impact of alcohol related harm on deprived communities.

Patient navigation was introduced in the North-East, to further enhance ACTs' success and to improve uptake of aftercare treatment in the community. While navigators are a novel role in the ACT, patient navigators roles have existed for some time outside of the UK. Roles were originally developed in the US in the 1990's and have since then been introduced in Europe across a range of settings [17]. Patient navigation is founded on a patient-centred model, and its central aim is to remove barriers to enable patients to access the care they need, when they need it [18]. Patient navigation has potential to contribute to mitigating health inequalities, although more research is needed [[19], [20], [21]]. In the US, a randomised controlled trial established a reduction in emergency care visits and costs when frequently attending patients, had access to a Patient Navigator [22].

Alcohol Recovery Navigators have been employed since 2021 by a drug and alcohol service. Funding was initially provided by the Integrated Care Board (ICB). The Navigators are managed by the Alcohol Care Team Lead who also provide clinical supervision. Their role is divided into two parts: 1) Alcohol Recovery Navigator in the hospital and 2) Alcohol Recovery Worker in the community. As hospital Alcohol Recovery Navigators, their primary task is to establish connections between the hospital and community for patients admitted with alcohol-related issues. As community Alcohol Recovery Workers, they are additionally responsible for ensuring that the hospital care patients receive, continues seamlessly in the community. To facilitate this bridging function, an agreement is in place to enable sharing of information between the Emergency Department (ED) and community services.

This qualitative study aimed to evaluate perceived impact of the Alcohol Recovery Navigator role and the programme's implementation by exploring perspectives of staff working as Navigators, or working closely with them as frontline care colleagues, or service managers. In addressing the gap in implementation research, this study offers valuable implications for healthcare delivery and policy for an underserved population group. This study lays the groundwork for future exploration of the effectiveness of Alcohol Recovery Navigators, helping to inform the refinement and long-term sustainability of alcohol-related care pathways across hospital and community settings.

## 2 Methods

This study was conducted in a single North-East NHS hospital Trust.

The study's guiding theoretical framework was Normalisation Process Theory (NPT), which offers a set of conceptual tools to understand how new interventions become embedded in routine practice and contribute to identifying conditions essential for effective implementation [23]. NPT outlines four core constructs relevant to implementing a new practice or intervention: 1) Coherence (individual or collective sense-making); 2) Cognitive participation (building and sustaining a community of practices); 3) Collective action (operationalising practices); and 4) Reflexive monitoring (assessing and understanding the impact of new practices on individuals and others) [24]. Examining implementation through these constructs helps reveal underlying mechanisms shaping people's efforts and using a dual approach allowed the retention of the richness of data and connecting experiences to theoretical constructs [25]. Our positionality as public health researchers with prior knowledge of implementation processes informed our use of NPT. This enabled us to situate emergent themes within a robust theoretical framework, ensuring the analysis remained theory-driven while grounded in participants' accounts.

As this study aimed to explore in-depth perspectives of staff who were working as or alongside Navigators, a qualitative study with a phenomenological approach was conducted [26]. The study was underpinned by a constructivist paradigm; the researcher acknowledged the subjective and socially constructed nature of participants’ own experiences. The Standards for Reporting Qualitative Research (SRQR) checklist was used to improve transparency [27].

Purposive sampling was used, supplemented by snowball sampling. Inclusion criteria stipulated participants should be: Navigators, working or having worked closely with Navigators, or having worked actively to support implementation of Navigators for a minimum of three months. Not everyone who was invited to participate chose to take part. Participants were initially approached through a gatekeeper, who circulated an email to relevant staff advertising the research and including a participant information sheet. Those interested in participating were asked to email the researcher (MC), and not the gatekeeper, to help reduce any potential pressure to participate. Eight participants were recruited, six from ED and two from community services, all participants were sent a consent form which was signed electronically and returned via email to the researcher. Participating staff included a consultant (n = 1), nurses (n = 3), community managers in alcohol care (n = 2) and Alcohol Recovery Navigators (n = 2); length of service ranged from 1 to 10 years.

Data collection methods consisted of semi-structured interviews, which were conducted over Microsoft Teams between June to July 2022 by MC, who was at the time a Public Health Practitioner at a different local authority and MSc student in Public Health. A topic guide (included as supplement) containing open-ended questions, was developed to delve deeply into various perspectives [26], with questions informed by NPT constructs. The topic guide included questions aimed at exploring participants' experiences with the Navigator role, how they were introduced to the programme, what their understanding of the role was, and participants’ reflections on how the role impacted patients. Interviews were audio recorded via Microsoft Teams. Credibility was ensured by actively checking understanding during interviews via clarification and paraphrasing. This approach is supported in qualitative methods as a valid method of member checking [28]. Transcripts were checked for errors post-interview by the researcher.

A two-stage process of analysis was undertaken. First, we conducted an inductive qualitative analysis, selected for its theoretical and methodological transparency [29]. This allowed themes to emerge directly from participants’ experiences, without reliance on a predefined framework. Once key themes were identified, we drew on NPT to support interpretation and situate the findings within a broader theoretical context [26]. No themes were omitted or modified during the mapping process. Data were coded and analysed by MC, with codes and themes subsequently reviewed by FC.

## 3 Results

Findings are presented following themes that were mapped onto the NPT constructs of coherence, cognitive participation, collective action, and reflexive monitoring (Table 1).

**Table 1:** Themes mapped to Normalisation Process Theory (NPT) constructs, with representative quotations.

NPT Constructs	Theme	Representative quotations
**Coherence:** the sense making people do individually or collectively	Staff understanding and knowledge of the Navigator role	*“Trying to find the balance between the two roles and understanding where one role ends, and another role starts, and I think you know that that's taken all of us probably a good six months to really understand the differences between the Navigator role and then then the role in [the community service].”* (Participant 003, Community Manager in alcohol care)
**Cognitive participation:** the work people do to build or sustain a community of practices of a new intervention	Staff engagement and commitment to the Navigator programme	*“I'm really passionate about supporting people in the recovery and I think you, you know, if we're gonna advocate for them, if we're gonna kind of support them and know that there's somebody looking out for them, whilst especially while they're in hospital because it could be, you know, quite a scary time for them if they've got no family networks or, you know, some people have nothing. And so it's been really on the plus side, we've built really good working relationships with the hospital staff now.”* (Participant 004, Alcohol Recovery Navigator)
**Collective action:** the operational work people do to enact a set of practices	Implementing the Navigator role to enable the work	“*It joins up that community and hospital like our service and the Community team much better. And we've got like obviously instant access to notes and work and mobile phone numbers. And I think it just makes that better relationship for us and better patient journey*.” (Participant 001, Nurse)
**Reflexive Monitoring:** the appraisal work people do to assess and to understand the ways a new set of practices affect them and others around them	Staff reflection on appraising the intervention and embedding change	“*It's really, really, really positive and bridges the gap between hospital and community really, really nicely. And I know a lot of people, a lot of patients are opening up a lot more when they see a familiar face and it's nice to, it eases any patients' anxieties known that they're gonna see the same person when they get discharged at the community services*.” (Participant 005, Community Manager in alcohol care).

### 3.1 Staff understanding of the Navigator role (coherence)

Regardless of their professional roles, all participants reported having undergone a period of uncertainty about the Navigator's role and responsibilities when it was introduced. Staff revealed they believed initial confusion stemmed from the split role between hospital and community service. Slight variations in the role description were noted by participants in the first six months of implementation. For example, participants reported that initially, individuals within the ACT and ED did not understand the difference between the Navigator role and a member of the ACT team. However, participants expressed that gradually understanding of the role improved. At time of interview, all participants, Navigators and non-Navigators, shared a similar fundamental understanding of the Navigator role's purpose: to support patients to access other hospital and community services based on their individual needs.

“Practically they provide like, one-to-one support for patients that are in hospital who are identified as being alcohol dependent or having alcohol-related admission and they do like a recovery programme and to support like what matters to the patients. So that could be like housing benefits, welfare and obviously the work with like the Community team and the hospital teams.” (Participant 001, nursing staff)

All participants shared that they thought the strategic overarching purpose of the role was to reduce hospital admissions and days spent in hospital. By offering those patients support, participants believed patients did not have to re-tell their stories, had assistance to connect back into wider support services and had an opportunity for more sustained engagement with drug and alcohol services. Participants recognised that Navigators could help build trusting relationships with those repeatedly admitted to hospital by dedicating time to support them and identify root causes of their alcohol-related admissions.

“*So you’re getting to know somebody and introducing yourself and then if they are comfortable with you, then we can work with them in the community. So it's like a smooth transition from hospital to community*” (Participant 007, Navigator).

### 3.2 Staff engagement and commitment towards the Navigator role (cognitive participation)

All participants expressed their support for the Navigator role. They discussed the level of empathy required to work with patients who often presented as “*chaotic*”. Participants shared that alcohol-dependent patients frequently return to ED, and they described how this can lead to frustration or exhaustion among some healthcare providers. Participants expressed a strong commitment to support those “*frequent attenders*” and give them the best possible care. Participants shared they encouraged other hospital employees not to dismiss patients’ needs, aiming to improve the overall patient experience.

Participants shared that they believed this patient group was often stigmatised and discussed times when other hospital staff had been dismissive of patients. Participants reported patient feedback had indicated this, and patients had reported to participants they had perceived themselves as a burden to hospital staff or felt they were not taken seriously. Participants described how other staff members in ED would now come to the Navigators for advice and support to engage with patients’ needs.

*“ I think a lot of our patients don’t have the best experience in hospital, so seeing the same person over again who works within the service, who has a passion for working with alcohol-dependent patients, and who can be an advocate for patients, it’s really, really beneficial. So they have a more positive hospital journey and hopefully prevent admissions as well. But with the support and place in the community.”* (Participant 002, nursing staff)

### 3.3 Implementing the Navigator role to enable the work (collective action)

Navigator participants described that initial implementation of the role took some time, mostly related to confusion about the role. Non-Navigator participants reported they were now working closely with Navigators and other services, building better communication to offer a more patient-centred approach. All participants believed that staff working with Navigators improved their engagement and relationships with patients. This improvement was attributed to the Navigators’ skills and knowledge of available services.

All participants explained that the ACT and community drug and alcohol service were integral components of existing services for patients admitted for alcohol-related reasons. Participants expressed that over time, as the role became more established, it aligned hospital-based treatment with the possibility of ongoing community-based treatment.

“*I think it's that interface between community services and the hospitals. So I don't think that we're always very good at linking between the two*” (Participant 008, Consultant).

Participants described how the Navigators' skills, in conjunction with their knowledge, improved treatment options offered to patients and how this facilitated development of stronger staff-patient relationships. They stated that patients had opportunities to develop trust with staff, while staff could consult Navigators and connect patients with other services. Because the Navigators work in both the community team and ACT, they have access to information systems in both settings. Participants shared that with an existing data-sharing agreement and collaboration across teams, Navigators facilitate a more seamless care transition and information sharing between hospital and community. Participants described how previously there were barriers in terms of communication between staff in acute to community settings. Participants discussed that since the Navigator role was introduced, relationships had improved which they believed enhanced communication, strengthened working relationships, and impacted positively on patients’ experiences.

*“It joins up that community, hospital [..] and the Community team much better* [..] *And I think it just makes better relationships [..] and better patient journey.” (Participant 001, nursing staff)*

Participants agreed that the only additional resource needed to improve the service is more staff. Participants felt that having only one full-time equivalent role might result in some patients being missed, particularly those admitted out of hours and weekends, or if patients were discharged while Navigators were not at work or were not notified of discharge.

*“I think it's the cover aspect, you know, to ensure that because one person can't cover however many sites in 24 hours and I think it if we had enough bodies to ensure that there was a 24-hour coverage, it would eliminate some of the pressure that's on ED staff and Ward staff in all honesty.” (Participant 005,* community manager in alcohol care*).*

### 3.4 Staff reflection on appraising the intervention and embedding change (reflexive monitoring)

Participants noted an increase in referrals to drug and alcohol treatment services and improved adherence rates, which they felt could result in potential positive outcomes for patients. Before the Navigator role, referrals were often incomplete and of poor quality; the introduction of the role was seen to improve both the quality and consistency of referrals, with clearer information. Participants shared that patients were now seen more quickly, often at the point of referral, thanks to Navigators and stated that staff can respond rapidly to referrals, enhancing patient engagement.

Participants also reported how they measured informally what success looks like in the Navigator programme. They felt that the work of the Navigator did not necessarily lead to patients becoming abstinent; rather participants viewed success as patients making smaller changes or having a willingness to engage in support. Participants discussed the extended timeframe it often takes for patients to acknowledge the harmfulness of their drinking patterns before fully engaging with support.

“I would guess my definition of success is I don't necessarily think it has to be abstinence. But I think a willingness to open up, a willingness to accept support, and even more so just an understanding that the support is out there, even if the patients don't choose to accept it at this point, just an awareness that it is here should they choose to accept it.” (Participant 006, nursing staff).

Participants acknowledged the difficulty of formally evaluating the Navigator role's impact, given that patients often present with multiple and complex health and social needs. This made it challenging to quantify how much the role directly contributed to outcomes, and some felt the current measurement approaches were insufficient. Even so, participants identified several positive outcomes, most notably fewer repeat hospital admissions and increased abstinence from alcohol. They also believed that some additional benefits may not be fully captured by current measures, underscoring the need for more comprehensive approaches to outcome evaluation.

## 4 Discussion

Timely allocation of Recovery Navigators was valued by hospital staff and perceived to support more seamless care. Participants described that Navigators reached patients with recurring alcohol-related admissions and helped to reduce these readmissions. Staff also associated the role with a better hospital journey for patients, particularly when Navigators were involved early in admission. Importantly, Navigators were seen as improving staff–patient relationships and facilitating continuity from emergency department through to structured treatment.

Clarity about the Navigator role was essential for successful implementation. Embedding the role in hospital practice took several months, with staff gradually developing an understanding of how to utilise it. This reflects findings from other studies, where confusion around roles and responsibilities has hindered service delivery and implementation [[30], [31], [32], [33]]. Using NPT allowed us to explore how professionals perceived the role's purpose and contribution. Participants understood the Navigator role as part of the Alcohol Care Team's overarching aim of reducing repeat alcohol-related admissions by supporting patients' social, physical, and mental health needs. This aligns with national priorities identified in Dame Carol Black's Harm to Hope report [34], which highlighted the need for greater integration of drugs and alcohol services within NHS and mental health provision.

**Embedding the Navigator role relied on clear definitions and joint working across professional groups.** Participants described the importance of intentional relationship-building and time to establish confidence in the role. Over time, the Navigator became integrated within the hospital's multidisciplinary approach to patient care, with staff readily collaborating and referring patients to wider services. This strong service delivery model contrasts with findings from other studies, which reported less successful integration and ongoing challenges in cross-system working [31,32,35]. For example, Beverly et al. (2018) highlighted the benefits of diabetes care Navigators but noted difficulties in achieving effective integration across systems [35]. Our findings therefore suggest that deliberate efforts to clarify roles and encouraging collaboration are central to embedding navigation services.

Effective collaboration and information sharing supported the embedding of the Navigator role across hospital and community services. Participants described how joint working and role clarification facilitated cross-disciplinary collaboration and strengthened links with wider community provision. This mirrors findings from other UK Navigator programmes [36] and supports Beverley et al.’s (2018) conclusion that joint working is critical to effective navigation [35]. In other settings, lack of information sharing has been identified as a barrier to implementation [29]. In contrast, our study benefited from existing data-sharing agreements and a shared role across hospital and community, which enabled Navigators and staff to collaborate more closely. This context is a key recommendation from our findings, as fragmented care and poor communication can lead to poorer outcomes and diminished trust in health services [37].

Recovery Navigators played a crucial role in maintaining continuity of care between hospital and community services. Participants highlighted that Navigators bridged gaps in patient journeys by improving communication across services and supporting access to community provision. Clear boundaries and collaboration with community services were described as essential to avoid duplication or confusion for patients [38]. This strength was partly attributed to the Navigator role's operation across both hospital and community settings. Comparable findings have been reported elsewhere: in Khapley et al.’s (2021) study of an HIV/AIDS Navigator programme in Australia, Navigators acted as a vital link in maintaining continuity between clinics and patients [39]. Similarly, Beverly et al. (2018) found that Navigators relayed patient information to staff and contributed to reducing disparities [35]. Our study adds to this literature by showing how trust, patient engagement, and streamlined referral processes enabled Navigators to sustain patient involvement during long waiting periods for community support.

Trust and relationship-building were central to the success of the Navigator role. Participants emphasised that Navigators' extensive knowledge of local services, combined with lived experience of alcohol recovery, enabled them to form meaningful relationships with patients. These qualities were seen as key requirements for staff in the role. A linked evaluation study found that patients valued Navigators’ gentle persistence and non-judgemental approach, which fostered trust and enabled honest conversations [38]. In our study, trust was not only central to patient–navigator relationships but also extended to interactions with other hospital staff, reinforcing integration within the care team. Building trust was described as a process of listening, responding to needs, and showing understanding. This finding is important given evidence that mistrust in health services can negatively affect engagement and outcomes [37,40].

Addressing inequality and stigma is essential to improving alcohol care, and Navigators were seen as helping to challenge these barriers. Economic inequality and structural stigma are recognised obstacles to delivering evidence-based alcohol interventions [41]. In a US evaluation, recruitment to a Navigator programme was hindered by self-stigmatisation among people with alcohol use [42]. Participants in our study felt that Navigators' empathic, patient-led approach encouraged patients to feel listened to and understood, and also helped shift attitudes among other hospital staff. In this way, the role was seen as contributing to the NHS's strategic goal of tackling health inequalities through holistic, patient-centred care [37].

This small qualitative study offers valuable insights into the implementation of an Alcohol Recovery Navigator role in the North-East of England. It adds to the growing evidence base regarding Navigator roles in the UK context, particularly relevant given the NHS Long Term Plan's (2019) emphasis on improving alcohol care [11]. Qualitative inquiry was deemed the most suitable approach due to the study's small sample size and single setting; a sufficiently powered quantitative analysis would have been unfeasible. Additionally, qualitative methods allowed for exploration of perspectives and nuances not captured by routine data collection. However, there are limitations to consider. Conducting the study within a single local setting restricted the pool of potential health and care staff participants with direct experience of the Navigator role, including both stakeholders and Alcohol Recovery Navigators themselves. This posed interpretation challenges, as some participants spoke from personal experience while others reflected from an external perspective, potentially contributing to variability in how the role was described and understood. Since the completion of this study, additional Navigators have been recruited across the region, and a more comprehensive evaluation has been undertaken across six hospitals, drawing on both qualitative and quantitative data. This larger study, using both qualitative and quantitative methods, provides a broader evidence base on implementation and impact, and complements the insights generated here [38]. Participants already held positive attitudes about the Navigator role and integrated alcohol care, potentially limiting the exploration of contrasting views. Additionally, the study did not include patients or their close social contacts, relying solely on staff statements about patient experiences. While conversations with patients and their support networks would have enriched the analysis, resource and time constraints prevented their inclusion in this project. The ongoing region-wide evaluation addresses this limitation by involving patients, carers, and healthcare professionals, building on the findings of this preliminary study. Although we were not able to access service-level data (e.g. uptake by gender or ethnicity), our qualitative findings offer important insights into perceptions and experiences of the service. Future studies should build on this by incorporating an intersectional lens and routinely collected demographic data to better understand for whom the service works best.

In conclusion, this qualitative study provides important insights into how Alcohol Recovery Navigators are perceived to impact care and how the role can be successfully implemented. Staff valued timely allocation of Navigators, describing how they bridged hospital and community services, improved continuity of care, and strengthened patient–staff relationships. Effective implementation was supported by role clarity, collaboration across professional groups, and existing data-sharing agreements. Navigators’ lived experience and non-judgemental approach were seen as central to building trust with patients and shifting staff attitudes, contributing to more patient-centred care. Importantly, participants highlighted that recovery should be measured holistically, with outcomes such as improved housing, family relationships, and empowerment recognised alongside abstinence and reduced admissions.

Future research should examine the effectiveness of Navigator roles using larger, multi-site samples, with attention to equity and intersectionality. Understanding for whom Navigator programmes work best, and under what circumstances, will be critical to ensuring their sustainability and impact on reducing alcohol-related harm.

## Ethical approval

Ethical approval was provided by the University of Sunderland's Research Ethics Group on 28/04/2022.

## Author contribution statement

All authors contributed to the critical review and development of this final manuscript for publication. MC conceived and designed the study under supervision of FC and RS. MC conducted data collection and data analysis, supported and facilitated by FC and RS. MC wrote the first draft of the manuscript under the supervision of FC, FC took responsibility for further drafts, supported by MC and DS, with senior oversight from AOD. RS, JC, SH, KJ, EJH all commented on versions of the manuscript.

## Funding

The study did not receive funding.

## Declaration of competing interest

The authors declare that they have no known competing financial interests or personal relationships that could have appeared to influence the work reported in this paper.

The author is an Editorial Board Member/Editor-in-Chief/Associate Editor/Guest Editor for *[Perspectives in Public Health]* and was not involved in the editorial review or the decision to publish this article.
